# Advance in molecular mechanisms underlying diabetes related to viral hepatitis infection

**DOI:** 10.3389/fcimb.2025.1661155

**Published:** 2025-08-22

**Authors:** Chuyan Wang, Miao Yu, Yilin Che, Ruyi Du, Yaoyao Xu, Junqi Niu, Xiumei Chi

**Affiliations:** ^1^ Core Facility of the First Hospital of Jilin University, Changchun, Jilin, China; ^2^ School of Public Health, Jilin University, Changchun, Jilin, China; ^3^ Hepatology, The First Hospital of Jilin University, Changchun, Jilin, China

**Keywords:** viral hepatitis, diabetes, oxidative stress, epigenetic modifications, inflammatory pathways

## Abstract

Diabetes and viral hepatitis, particularly hepatitis B (HBV) and hepatitis C (HCV), are significant global health burdens with complex interconnections. This review discusses the molecular mechanisms linking viral hepatitis to diabetes, focusing on inflammatory pathways, oxidative stress, and epigenetic modifications. Key findings highlight the role of STAT3 in promoting insulin resistance and β-cell apoptosis, the impact of ER stress and NOX-mediated oxidative stress on metabolic dysfunction, and the influence of epigenetic changes such as DNA methylation and histone acetylation on glucose homeostasis. These interconnected pathways provide insights into the pathogenesis of diabetes in hepatitis patients and suggest potential therapeutic targets for managing these co-occurring conditions. Future research directions include exploring the synergistic effects of these pathways and leveraging advanced technologies for personalized treatment strategies.

## 1 Background

Viral hepatitis, caused by multiple hepatitis viruses, remains a significant global health challenge, with hepatitis B virus (HBV) and hepatitis C virus (HCV) posing particular threats due to their propensity for chronic infection and severe clinical outcomes such as liver cirrhosis and hepatocellular carcinoma. According to 2022 epidemiological data, approximately 257 million people worldwide live with chronic HBV infection. Together, HBV and HCV account for an estimated 1.3 million annual deaths, ranking viral hepatitis as the second leading cause of infectious disease mortality after Corona Virus Disease 2019 (Burki, 2024). While HCV-related mortality has declined significantly in recent years following the introduction of highly effective direct-acting antivirals (DAAs) (Cui et al., 2023), the absolute burden of HBV-related diseases continues to rise, driven by demographic factors including population growth and aging (Collaborators, G.H.B, 2022).

Concurrently, diabetes mellitus, a metabolic disorder characterized by chronic hyperglycemia, exhibits a persistently rising global prevalence. Recent epidemiological projections estimate that 642 million individuals aged 20–79 currently live with diabetes worldwide (Ogurtsova et al., 2017). Of particular clinical significance, mounting evidence demonstrates a strong association between viral hepatitis (predominantly HBV and HCV infections) and diabetes pathogenesis. A large-scale cohort study involving 55,520 participants established that chronic HBV carriers and individuals with prior HBV exposure (defined as hepatitis B surface antigen (HBsAg)-negative/hepatitis B core antibody (HBcAb)-positive serostatus) face substantially elevated diabetes risk (Lei et al., 2020). A study demonstrated that chronic hepatitis B patients with impaired fasting glucose experience worsening islet function due to liver fibrosis progression, consequently elevating their diabetes risk (Lu et al., 2023). Epidemiological evidence supports a bidirectional association between diabetes and HCV infection. Notably, some research suggests diabetes could serve as an extrahepatic complication of chronic HCV infection (Naderi et al., 2025). By histological evaluation of the pancreas in HCV seropositive patients, it was observed that HCV can directly lead to the morphological and functional defects in pancreatic beta cells. Significant reduction in ΔC-peptide 30 and insulinogenic index also demonstrated the HCV-induced pancreatic dysfunction via direct effect on beta cells (Chen et al., 2024). Although HCV can now be cured by DAAs, there are mixed voices regarding whether this treatment is associated with a reduced risk of diabetes onset. Some studies suggest that patients who achieve sustained virologic response have a decreased risk of developing T2DM and a corresponding reduction in insulin resistance (Jabeen et al., 2025). However, other research indicates that DAAs treatment is not related to the reduced risk of diabetes occurrence, which might be associated with excessive weight gain after DAAs treatment (Jeong et al., 2025).

While the association between HBV/HCV infection and diabetes has been well-documented, the underlying molecular mechanisms remain incompletely understood. This review systematically examines established pathways including insulin resistance and hepatic inflammation, while also investigating emerging areas such as epigenetic regulation. The ultimate goal is to translate fundamental laboratory discoveries into clinically applicable biomarkers for disease monitoring, thereby offering novel therapeutic strategies to prevent diabetes progression and manage complications in viral hepatitis patients.

## 2 Inflammatory/immune pathway

### 2.1 Inflammatory pathways caused by STAT3

The family of Signal Transducer and Activator of Transcription (STAT) proteins, which includes STAT1, STAT2, STAT3, STAT4, STAT5a, STAT5b, and STAT6, functions as cytoplasmic transcription factors that mediate various intracellular signaling pathways. Notably, STAT3 plays a pivotal role in several biological processes, including cell proliferation, survival, differentiation, and angiogenesis (Zou et al., 2020).

According to the canonical mechanism, inactive STAT3 resides in the cytoplasm. Upon stimulation by growth factors, cytokines, hormones, or other oncogenic proteins, the upstream proteins subsequently induce the phosphorylation of STAT3 at tyrosine 705 (Y705) and serine 727 (S727) residues in the C-terminal transactivation domain (Wang et al., 2024). This phosphorylation triggers STAT3 dimerization and subsequent nuclear translocation. In the nucleus, STAT3 binds to DNA to promote transcription of target genes (Chen et al., 2023).

#### 2.1.1 STAT3 causes beta cell necrosis by IFN-γ

STAT3 is a key factor contributing to HBV-induced hepatic inflammation activation. Research has determined that the continuous expression of hepatitis B virus X protein (HBx) leads to the activation of the Janus kinase (JAK)/STAT signaling pathway (Lee and Yun, 1998). HBx, a compact protein consisting of 154 amino acids, serves as a multifaceted regulator. It modulates an array of host processes by either directly or indirectly interacting with both viral and host factors (Zhao et al., 2024). This activation of STAT3 resulted in a significant increase in acute inflammatory factors such as serum amyloid A1, Intercellular adhesion molecule 1, S100a8/9/11, and C-X-C motif ligand 1/14 (CXCL1/14), among others, which was subsequently followed by a substantial surge in monocytes/macrophages and a moderate increase in T cells within the liver (Tang et al., 2024). In addition to the role of HBV, STAT3 can also be activated by HCV. HCV core protein directly interacts with and activates STAT3 through phosphorylation of the critical tyrosine residue (Zhao et al., 2016).

HBV-induced liver cirrhosis patients exhibit elevated levels of S100A8/A9, natural killer (NK) cells, and interferon-gamma (IFN-γ) following STAT3 activation, a biomarker profile associated with diabetes development (Li et al., 2024). Mechanistically, S100A8/A9 engages the receptor for advanced glycation end products (RAGE) to activate p38 mitogen-activated protein kinases (p38 MAPK), thereby stimulating IFN-γ secretion (Li et al., 2024). This pathway establishes a molecular bridge between viral hepatitis and diabetes. As a multifunctional pattern-recognition receptor, RAGE binds diverse endogenous ligands and triggers downstream inflammatory cascades such as JAK/STAT signaling. Notably, S100A8/A9-RAGE interaction reciprocally activates the JAK2/STAT3 pathway (Xin et al., 2024). Furthermore, S100A8/A9 promotes IL-6 and receptor activator of nuclear factor kappa-B ligand (RANKL) production in osteocytes through coordinated RAGE and Toll-like receptor 4 (TLR4) signaling (Takagi et al., 2020). This study uncovers a regulatory paradox where IL-6, conventionally recognized as a primary STAT3 activator, is itself upregulated by S100A8/A9 through STAT3-dependent mechanisms, establishing a positive feedback loop (Böttcher et al., 2022). The S100A8/A9-p38 MAPK axis elevates IFN-γ production in natural killer (NK) cells (Li et al., 2024), subsequently inducing β-cell necroptosis and insulin deficiency through mechanisms mirroring those of classic pro-inflammatory cytokines, including tumor necrosis factor-alpha (TNF-α) and IL-1β from infiltrating immune cells (Sun et al., 2020; Li et al., 2024). Furthermore, IFN-γ synergizes with LIGHT to activate β-cell apoptosis via the nuclear factor kappa-B NF-κB/B-cell lymphoma/leukemia 2 Bcl2 pathway (Zheng et al., 2016). Thus, hepatitis viruses drive IFN-γ-mediated β-cell apoptosis through STAT3/IL-6/S100A8/A9 crosstalk, promoting insulin resistance and diabetes risk.

#### 2.1.2 STAT3 induces Socs-mediated insulin resistance

Hepatitis viruses can induce the expression of suppressor of cytokine signaling (SOCS), a group of proteins that negatively regulate cytokine receptor signaling via the JAK/STAT pathway (Sobah et al., 2021), thereby disrupting insulin signaling and promoting insulin resistance. Specifically, HBV proteins such as HBx can synergize with CCAAT/enhancer-binding protein α(C/EBPα) to activate the STAT3-SOCS3 pathway, upregulating SOCS3 expression. While hepatitis B e antigen (HBeAg) and hepatitis B surface antigen (HBsAg) also modulate SOCS expression through distinct mechanisms (Xie et al., 2021). Similarly, multiple HCV proteins (e.g., core protein and p7) can induce SOCS1/3/7 expression via STAT3-dependent or -independent pathways, such as extracellular signal-regulated kinase (ERK) or peroxisome proliferator-activated receptor γ (PPAR-γ) (Pazienza et al., 2010; Convery et al., 2019). Notably, elevated hepatic SOCS3 expression in genotype 1-infected patients may contribute to obesity-associated interference with IFN-α biological responses (Walsh et al., 2006).

**Figure 1:**
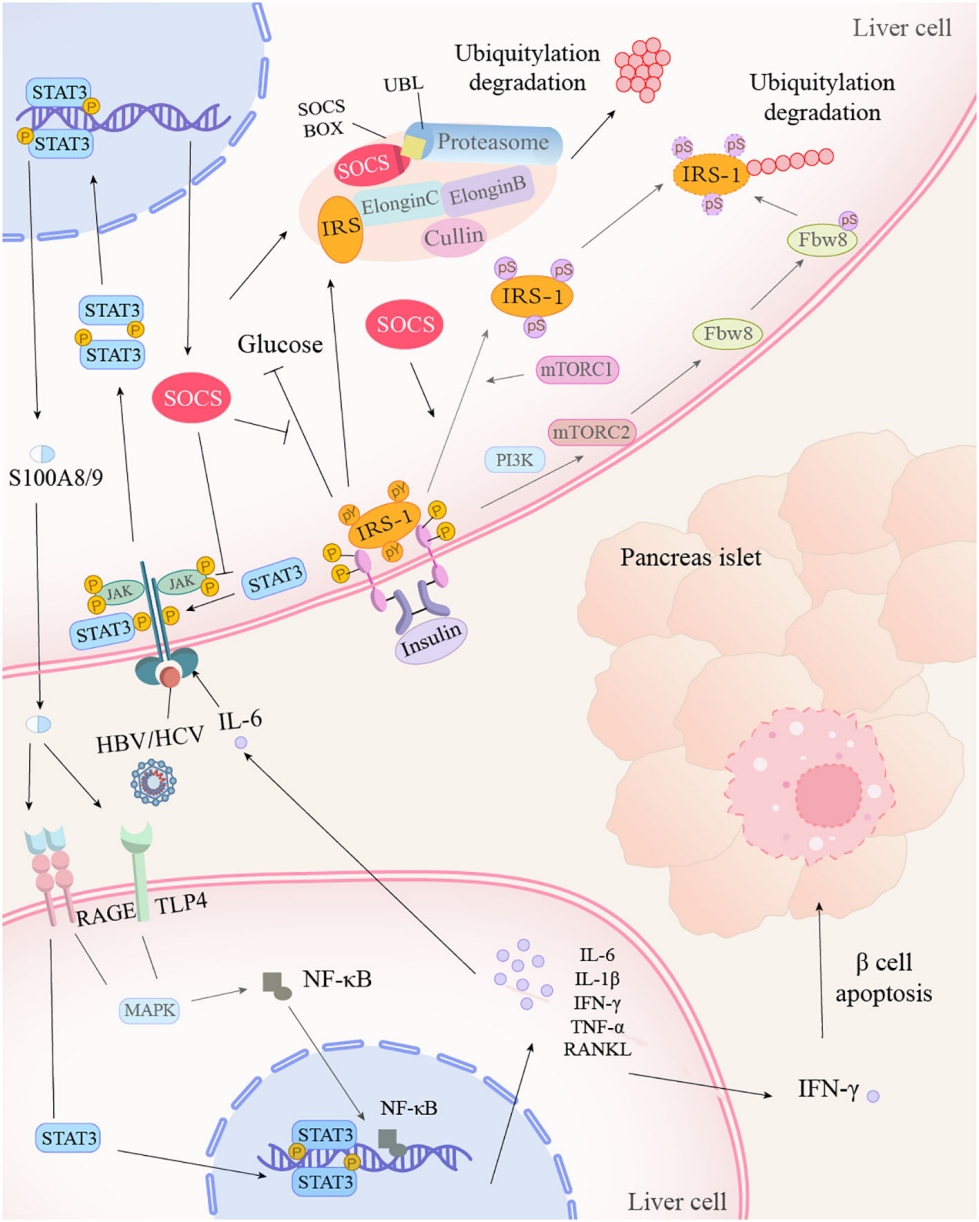
Hepatitis virus promotes insulin resistance through the STAT3 inflammatory pathway. After hepatitis virus or IL-6 acts on the receptor, STAT3 is phosphorylated and activated by JAK kinases, subsequently forming dimers. The STAT3 dimer translocates into the nucleus, where it binds to target genes, promoting the expression of S100A8/9 and its inhibitor, SOCS. SOCS, on one hand, inhibits the blood glucose-lowering process of IRS-1, which is combined with insulin. On the other hand, it collaborates with the proteasome to mediate the ubiquitination and degradation of IRS. Additionally, SOCS facilitates the conversion of tyrosine to serine on IRS-1, which, under the action of mTOR and Fbw8, also leads to IRS-1 degradation. The intracellularly produced S100A8/9 is transported extracellularly and enters other cells via the RAGE and TLR4 receptors, activating the STAT3 and MAPK signaling pathways, as well as NF-κB production. Under the influence of STAT3 and NF-κB, cells produce numerous inflammatory cytokines. Among them, IFN-γ induces apoptosis of β-cells.

In cultured cells and animal tissues, SOCS1/3 promote insulin resistance by facilitating the ubiquitination and degradation of insulin receptor substrates (IRS1/2). SOCS3 directly binds IRS1/2 and recruits the elongin BC ubiquitin ligase complex, together with the Elongin B ubiquitin-like protein (UBL) and Cullin-2, thereby enhancing IRS1/2 ubiquitination and degradation (Kamura et al., 1998; Rui et al., 2002; Babon et al., 2008). The current data are still consistent with the notion that Ser/Threonine phosphorylation of IRS-1, induced by a rapamycin-sensitive pathway through the mechanistic target of rapamycin complex 1(mTORC1), may impair insulin-stimulated tyrosine phosphorylation of IRS-1, which negatively regulates insulin signaling (Nguyen et al., 2022). IL-6-induced SOCS inhibits the tyrosine phosphorylation pathway and ultimately promotes the proteasomal degradation of IRS (Li et al., 2022). Additionally, it stabilizes F-box and WD repeat domain-containing protein 8 (Fbw8) via phosphorylation at Ser86, enabling insulin-induced cytoplasmic translocation of Fbw8 and subsequent IRS-1 degradation (Kim et al., 2012). Furthermore, HBx and SOCS3 can antagonize insulin-mediated suppression of gluconeogenesis by activating the promoters of phosphoenolpyruvate carboxykinase (PEPCK) and glucose-6-phosphatase (G6Pase) (Sharabi et al., 2017; Xiao et al., 2022). Collectively, these findings suggest that hepatitis viruses promote insulin resistance and type 2 diabetes mellitus (T2DM) through STAT3-dependent mechanisms, including IRS functional inhibition, ubiquitin-mediated degradation, and metabolic reprogramming (**Figure 1**).

HBV hepatitis B virus, HCV hepatitis C virus, IL-1β/6 interleukin-1β/6, STAT signal transducer and activator of transcription, JAK janus kinase, SOCS suppressors of cytokine signaling, IRS insulin receptor substrate, mTORC1/2 mechanistic target of rapamycin complex 1/2, Fbw8 f-box and wd repeat domain containing 8, RAGE receptor of advanced glycation endproducts, TLR4 toll-like receptor 4, MAPK Mitogen-Activated Protein Kinases, NF-κB nuclear factor kappa-B, IFN-γ interferon gamma, PI3K phosphatidylinositol 3-kinase, RANKL nuclear factor kappa-B ligand, TNF-α tumor necrosis factor alpha, UBL ubiquitin-like protein.

### 2.2 The immunoregulatory role of TRAIL/TRAIL-R in inflammation

TNF-related apoptosis-inducing ligand (TRAIL), a member of the TNF superfamily, is capable of initiating caspase-8-dependent apoptosis in transformed cells, inducing apoptosis in a broad range of cancer cells (Lv et al., 2025a). Significantly, it achieves this without causing fatality in essential normal cells. This process is triggered through its binding and subsequent activation of two specific receptors: TRAIL-Receptor1 (TRAIL-R1) and TRAIL-R2 (Lv et al., 2025a), whereas TRAIL-R3 can inhibit TRAIL-induced apoptosis by competing with TRAIL for binding to death receptors such as TRAIL-R2 (Degli-Esposti et al., 1997). TRAIL plays a significant role in inhibiting HBV. TRAIL not only inhibited HBV replication via blocking DNA synthesis (Suehiro et al., 2023), but also suppressed the proliferation of autoreactive T-cells by inhibiting the production of IL-2, IL-4, and INF-γ, impeding cell cycle progression from the G1 phase to the S phase and restricting calcium influx (Koliaki and Katsilambros, 2022). In the inflamed HBV-infected liver, the notable presence of activated NK cells with elevated TRAIL levels could serve a dual purpose. On the one hand, while it is true that this mechanism may contribute to the death of hepatocytes infected with HBV, it can also result in non-specific damage to hepatocytes and exacerbate liver inflammation. On the other hand, it facilitates the apoptosis of T cells that express elevated TRAIL receptors. This process could potentially result in the depletion of HBV-specific T cells and compromise the immune system’s ability to eliminate HBV (Peppa et al., 2013). This apoptotic process requires the participation of TRAIL-R2. In HBV infection, HBV-specific CD8^+^T cells, especially those infiltrating the liver, overexpress the TRAIL-R2 death-inducing receptor, thereby delivering an apoptotic signal (Peppa et al., 2013). The upregulation of TRAIL-R2 expression can predispose cells to apoptosis triggered by TRAIL, through receptor-ligand interactions and subsequent signal transduction pathways. Besides TRAIL-R2, the HBV (HBx) influences TRAIL-R3 transcription via the activation of NF-κb signaling pathway. An increase in TRAIL-R3 can impede TRAIL-dependent apoptosis and TRAIL-mediated HBV replication inhibition within HBV-infected liver cells (Suehiro et al., 2023).

TRAIL may suppress autoimmune diseases by downregulating immune responses (Cardoso Alves et al., 2021), whereas TRAIL deficiency exacerbates autoimmune diabetes and enhances autoimmune responses, as demonstrated by experimental observations (Lamhamedi-Cherradi et al., 2003). TRAIL exerts its function by suppressing IL-2 production and cell cycle progression, thereby inhibiting the proliferation of diabetogenic T-cells in nonobese diabetic mice during the development of autoimmune type 1 diabetes (Mi et al., 2003). TRAIL inhibits pancreatic β-cell apoptosis in type 1 diabetes mellitus by enhancing tissue inhibitor of metalloproteinase 1 function. This mechanism may reduce matrix metalloproteinase activity (Fogarasi and Dima, 2024). Following TRAIL overexpression, subsequent protein kinase B (Akt/PKB) activation stimulates β-cell proliferation, ultimately producing an anti-diabetic effect that is associated with increased SOCS1 expression (Koliaki and Katsilambros, 2022; Fogarasi and Dima, 2024). In T2DM, TRAIL demonstrates beneficial effects through its immunosuppressive and immunoregulatory properties, which mitigate the chronic inflammation characteristic of T2DM. It exhibits proliferative action on the beta-cell mass of pancreatic islets, insulin-sensitizing and myogenic action on skeletal muscle tissue, and hepatoprotective action, mainly improvement of non-alcoholic fatty liver disease (NAFLD) (Koliaki and Katsilambros, 2022).

In summary, TRAIL represents a critical molecular nexus between hepatotropic viral infections and diabetes. It modulates the progression of HBV infection through immunoregulation and apoptosis control, while concurrently demonstrating pleiotropic effects in glucose metabolism and immune homeostasis. These dual properties position TRAIL as a promising therapeutic target for both disease entities. However, the mechanistic complexity of TRAIL signaling necessitates further rigorous investigation to optimize its therapeutic potential while mitigating potential adverse effects.

## 3 Oxidative stress

### 3.1 ER stress

**Figure 2:**
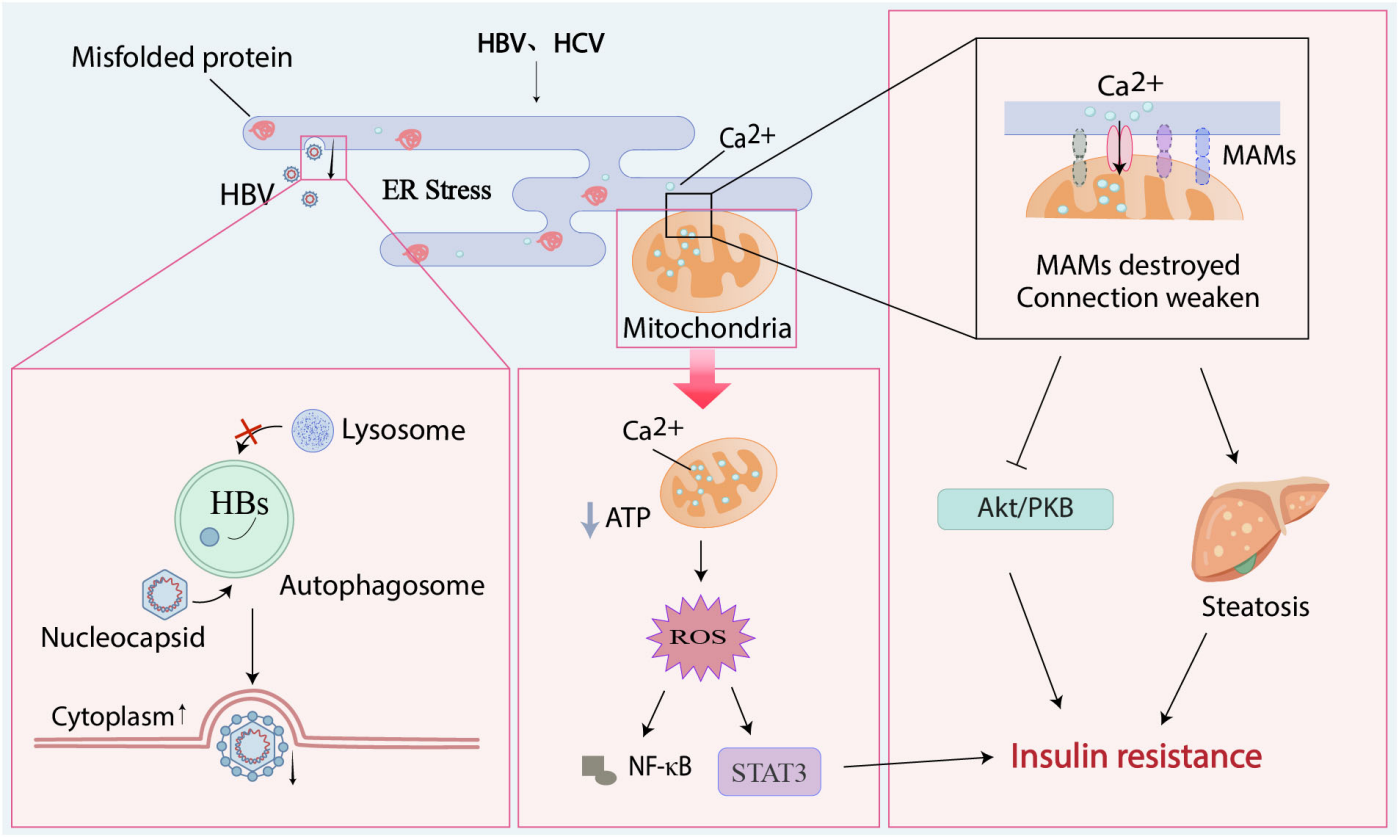
Endoplasmic reticulum stress plays a negative role in viral hepatitis and metabolic processes. Hepatitis viruses can cause the accumulation of unfolded proteins in the ER, leading to ER stress. Viral nucleocapsids enter autophagosomes, associate with HBsAg to form enveloped virions, block autophagosome-lysosome fusion, and are ultimately released extracellularly; the large amount of calcium ions entering the mitochondria from the stressed ER leads to the secretion of reactive oxygen species, which activates the STAT3 pathway and the secretion of NF-κB; the MAMs formed by mitochondria and ER are destroyed by this stress, and the channel proteins between them are missing, leading to the inhibition of the Akt/PKB signaling pathway and hepatic steatosis. These phenomena, together with STAT3, promote the occurrence of insulin resistance.

Endoplasmic reticulum (ER) stress refers to the cellular response when faced with various stresses or stimuli, characterized by the accumulation of misfolded and unfolded proteins within the ER lumen, as well as the disruption of calcium ion balance, which will happen in HBV-infected cells (Soh et al., 2024; Ghionescu et al., 2025). The HBx triggers ER stress through multiple mechanisms, including cellular ATP depletion, upregulated stromal cell-derived factor-1 expression, and exacerbated hepatic lipid accumulation, collectively enhancing autophagosome formation to facilitate viral replication and envelopment (Hu et al., 2022; Wang et al., 2022). Similarly, HCV infection elevates ER stress by impairing calcium regulation. Viral gene expression augments mitochondrial reactive oxygen species (ROS) production through calcium-dependent signaling pathways (Tardif et al., 2005). This oxidative stress in the liver is upregulated by not only the core nucleocapsid protein of HCV, but also other proteins such as NS3, NS5A, and NS5B (Martin de Fourchambault et al., 2023). The HCV nonstructural proteins NS3 through NS5B coordinate viral replication through formation of a ribonucleoprotein complex associated with ER-derived membranous webs, a process that can induce ER stress (Tardif et al., 2005). Notably, NS5A specifically activates both NF-κB and STAT-3 through mechanisms requiring coordinated Ca2^+^ signaling and reactive oxygen species (ROS) production from ER-mitochondrial crosstalk (Gong et al., 2001). Mitochondria and ER networks maintain intricate junctions, termed mitochondria-associated ER membranes (MAMs), which are instrumental in Ca2+ signaling, lipid trafficking, energy metabolism, and cell survival. However, in the process of hepatitis virus self-replicating and damaging host cells, it also destroys the MAMs by producing ER stress. MAMs represent a novel site for insulin-stimulated PKB/Akt phosphorylation. Compromised MAM integrity may impair insulin signaling pathways (including Akt/PKB activation) and disrupt glucose/lipid homeostasis, ultimately contributing to hepatic insulin resistance (IR). This mechanistic link suggests that hepatitis viruses may induce IR through oxidative stress-mediated MAM dysfunction (Tubbs et al., 2014). The pathways through which hepatitis virus-mediated ER stress may contribute to the development of diabetes are illustrated in the figure below (**Figure 2**).

Akt/PKB protein kinase B, ER endoplasmic reticulum, MAMs mitochondria-associated ER membranes, NF-κB nuclear factor kappa-B, HBs hepatitis B surface antigen, HBV hepatitis B virus, HCV hepatitis C virus, ROS reactive oxygen species.

### 3.2 NADPH oxidases

In addition to ER stress, hepatitis viruses can also mediate oxidative stress and cause inflammation through Nox. In HBV infection, clinical observations demonstrate significant reductions in serum glutathione and superoxide dismutase levels (Yang et al., 2020). The resulting HBV-induced ROS accumulation promotes hepatic inflammation through IL-6 upregulation in hepatocellular carcinoma (HCC), concurrently driving constitutive STAT3 activation. Regarding HCV, the NS3 protein activates NADPH oxidase, which may contribute to the natural progression of hepatitis C infection (Matuz-Mares et al., 2022). Additionally, HCV infection induces sustained upregulation of Nox1/4, with Nox4 showing increased nuclear localization in both *in vitro* models and human liver tissues. These findings identify Nox1/4 as persistent endogenous ROS source during HCV pathogenesis (de Mochel et al., 2010). NOX overexpression under pathological conditions mediates ROS generation, commonly termed oxidative burst (Sandoval et al., 2018). This process activates multiple pathogenic pathways, including aldose reductase, advanced glycation end product formation, protein kinase C signaling, and hexosamine flux. Concurrently, it stimulates pro-inflammatory cytokines (TGF-β, TNF-α, NF-κB, IL-6, IL-18), endothelial growth factor production, hyperlipidemia development, and excessive collagen deposition (Laddha and Kulkarni, 2020). This oxidative stress from NOX may also contribute to insulin resistance. Although mitochondria are classically regarded as the major source of free radicals, emerging evidence indicates that NOX significantly contributes to mitochondrial ROS generation in β-cells. This NOX-derived ROS exacerbates β-cell dysfunction by impairing insulin secretion and action, primarily through oxidative damage to cellular components, including DNA, proteins, and lipids (Elumalai et al., 2021).

Beyond the aforementioned ROS-generating pathways, nuclear factor erythroid 2-related factor 2 (Nrf2) serves as a critical regulator of redox homeostasis in both viral hepatitis and diabetes. HCV-mediated suppression of Nrf2-antioxidant response element-regulated genes increases reactive oxygen intermediates, compromising host genome stability and regenerative capacity while promoting HCV pathogenesis (Bender and Hildt, 2019). This process concurrently activates c-Jun N-terminal kinase (JNK), leading to impaired insulin signaling required for hepatic regeneration (Bender and Hildt, 2019). Notably, Nrf2 deficiency in HCV-infected cells downregulates insulin receptor expression, establishing a direct link to insulin resistance (Barthel et al., 2016). HBV similarly modulates Nrf2 activity but through distinct mechanisms. HBV upregulates Nrf2 to enhance insulin receptor biosynthesis while inducing α-taxilin overexpression, which traps insulin receptors intracellularly. This dual effect reduces membrane receptor availability and insulin binding capacity in hepatocytes, as demonstrated in both experimental models and clinical observations (Barthel et al., 2016). Collectively, hepatitis viruses exploit ER stress and oxidative stress pathways to induce insulin resistance and metabolic dysregulation. The NOX-Nrf2 axis emerges as a central coordinator of oxidative damage, inflammatory activation, and insulin signaling impairment, mechanistically connecting viral persistence to diabetes development. These findings reveal novel therapeutic opportunities targeting oxidative stress responses and Nrf2 pathway modulation.

## 4 Epigenetics

### 4.1 PGC-1α related to phosphorylation

Peroxisome proliferator-activated receptor-γ coactivator 1-alpha (PGC-1α) functions as a master transcriptional regulator governing mitochondrial biogenesis and functional integrity, including oxidative phosphorylation capacity and ROS detoxification pathways (Rius-Pérez et al., 2020). In viral hepatitis pathogenesis, PGC-1α demonstrates critical regulatory functions. HBV infection triggers silent information regulator 1 (SIRT1) -mediated deacetylation of PGC-1α, which potentiates PPARα transcriptional activity. This molecular cascade ultimately enhances HBV transcriptional activity and viral replication, as validated across experimental models and clinical specimens (Monroy-Ramirez et al., 2021). Yao et al. demonstrated that HCV infection induces ER stress and upregulates both wild-type PGC-1α and its liver-specific PGC-1α. This upregulation enhances HCV replication while concurrently increasing phosphorylated cAMP-response element-binding protein (CREB) levels (Qian et al., 2024). Mechanistically, wild-type PGC-1α expression is regulated by CREB phosphorylation, whereas the liver-specific PGC-1α is modulated through CREB phosphorylation combined with forkhead box O1 (FoxO1) dephosphorylation (Yao et al., 2014). In addition, FOXO1 impedes PGC-1α transcription through its competition with CREB for the binding sites on transcriptional coactivators CREBBP/EP300 (Qian et al., 2024). HCV-mediated upregulation of PGC-1α exhibits tissue-specific metabolic regulatory effects. Current evidence demonstrates that in both human and animal models of T2DM, the expression of PGC-1α and its downstream target genes - which are critically involved in mitochondrial biogenesis and oxidative phosphorylation - is significantly reduced in skeletal muscle and adipose tissue, while being markedly elevated in the liver of diabetic mice (Qian et al., 2024). This tissue-specific expression pattern generates opposing metabolic consequences, with hepatic PGC-1α overexpression increasing glucose output through enhanced gluconeogenesis, whereas skeletal muscle PGC-1α improves glucose utilization by augmenting oxidative capacity (Qian et al., 2024). PGC-1α precisely controls glucose disposal as it’s involved in multiple glucose metabolic processes. For example, it raises muscle glycogen stores by suppressing glycolytic flux and downregulating related enzymes’ expression (Lim et al., 2024). In adipose tissue, diminished PGC-1α levels and impaired insulin signaling molecules correlate with metabolic dysfunction, further aggravating systemic insulin resistance in affected subjects (Qian et al., 2024). Existing evidence demonstrates that hepatitis virus-mediated diabetes progression involves tissue-specific PGC-1α upregulation. For PGC-1α to serve as either a biomarker for diabetes susceptibility or a therapeutic target for glycemic control, precise identification of its tissue-specific actions is essential to mitigate the virus-induced adverse effects on glucose metabolism.

### 4.2 DNMTs related to methylation

DNA methylation represents a fundamental epigenetic mechanism essential for transcriptional regulation, developmental processes, genomic imprinting, genome stability maintenance, and chromatin organization. This modification is precisely controlled by DNA methyltransferases (DNMTs), methyl-CpG binding domain proteins, and chromatin remodeling factors (Lee et al., 2024). In virally induced malignancies, viral oncoproteins can dysregulate the host methylation machinery through aberrant DNMTs activation, consequently increasing oncogenic risk (Mehdipour et al., 2020). Among DNMT family members, DNMT3A and DNMT3B demonstrate particular pathological significance in hepatitis virus infection. These *de novo* methyltransferases preferentially target unmethylated DNA substrates, establishing methylation patterns that contribute to viral pathogenesis (Lee et al., 2024). HBx inhibited the expression of protein tyrosine phosphatase non-receptor 13 (PTPN13) by elevating the expression levels of DNMT3A and establishing an interaction with DNMT3A (Dandri, 2020). PTPN13 can significantly inhibit HCC cell proliferation, migration, and invasion, and its overexpression can slow down the progression of HCC cells (Zhan et al., 2016; Dandri, 2020; Yan et al., 2021). Mechanistically, DNMT3A binds to the PTPN13 promoter and induces methylation-mediated silencing. These findings suggest that HBV may promote HCC development via DNA methylation-dependent pathways. The virus-induced elevation of DNMTs levels exhibits dual effects: while hypermethylation of host genes (e.g., PTPN13) disrupts normal cellular functions, methylation of viral DNA may paradoxically suppress HBV replication. Notably, hepatocytes upregulate DNMTs expression as a defensive response against HBV infection (Oropeza et al., 2021). These enzymes can methylate the HBV viral DNA, resulting in reduced viral replication and diminished viral gene expression. However, conflicting evidence exists regarding HBV covalently closed circular DNA (cccDNA) levels during DNMT3A expression. This phenomenon may be linked to the suppression of host factors that normally inhibit cccDNA formation under basal conditions (Brezgin et al., 2019). The upregulation of DNMTs such as DNMT1 and DNMT3b is also linked to HCV core protein (Hassouna et al., 2020), while there is no change in genotypes 2a, 3a, 4h, and 5a except in genotype 1b HCV (Benegiamo et al., 2012).

DNA methylation demonstrates a potential association with diabetes pathogenesis. Comparative analyses reveal elevated DNA methylation levels in the PPAR gamma coactivator 1-alpha (PGC-1α) gene promoter in pancreatic islets from diabetic subjects relative to non-diabetic controls. This epigenetic modification likely suppresses PGC-1α expression, subsequently contributing to hormonal imbalance, dysregulated lipid metabolism, and mitochondrial impairment, ultimately disrupting glucose homeostasis. These findings collectively support a mechanistic link between DNA methylation and diabetes development (Santos et al., 2020). DNA methylation inhibits the expression of the insulin gene, and the promoter of this gene is specifically demethylated in cells responsible for insulin production (Wu et al., 2023), associating variations in DNA methylation with overall body insulin sensitivity. Thus, researchers have already successfully demonstrated the therapeutic role of DNMTs in diabetes. DNMTs inhibitor can improve obesity-induced glucose intolerance and insulin resistance in a manner contingent upon adiponectin (Wang et al., 2023). In addition, someone discovered that deletion of DNMT3A specifically in the adipose tissue of mice protects from DNMT3A-induced insulin resistance (You et al., 2017). Meanwhile, DNMT3A inhibits fibroblast growth factor 21 (Fgf21), an insulin sensitizer (Markan et al., 2014), by elevating the DNA methylation status of the promoter region of Fgf21, which decreases Fgf21 expression, and suppresses its crucial negative regulatory target gene effect in adipocytes (You et al., 2017). Besides, two major epigenetic silencing mechanisms are instrumental in causing the functional immaturity observed in Raptor-deficient β-cells: DNMT3A-dependent DNA methylation and Polycomb repressive complex 2-dependent H3K27me3 modification (Ni et al., 2022). In conclusion, overexpression of DNMTs induced by the hepatitis virus may lead to diabetes susceptibility through epigenetic pathways. Future research can be focused on which genes related to insulin signaling are suppressed by the upregulated DNMTs expression, whether it affects the function of pancreatic β-cells and insulin sensitivity, and the specific impact of DNMTs inhibitors in both diseases.

### 4.3 HDACs and HATs related to acetylation

Histone acetylation, among the earliest identified post-translational modifications of nuclear proteins, serves fundamental structural and functional roles within chromatin. This epigenetic modification dynamically regulates diverse cellular processes spanning transcriptional activation, metabolic programming, proliferation-apoptosis balance, chromatin architecture maintenance, and DNA repair mechanisms (Miziak et al., 2024; Lv et al., 2025b). The counterbalancing deacetylation process, mediated by histone deacetylases (HDACs), equally governs transcriptional regulation. HDAC enzymes catalyze lysine deacetylation on both histone and non-histone substrates, inducing chromatin compaction and subsequent transcriptional silencing of target genes (Lv et al., 2025b). Existing studies have documented reduced expression of both HDAC3 mRNA and protein in HepG2 cells upon HBV infection (Wei and Meng, 2023). Given the established role of histone deacetylation in viral regulation, this observation raises the hypothesis that HBV may exploit HDAC3 downregulation to evade host antiviral defenses, thereby facilitating viral persistence through impaired deacetylation-mediated control of HBV replication. Therefore, research has confirmed that the expression of HDAC3 in HBV-infected mice is relatively weak. Furthermore, evidence suggests that reestablishing this particular signature can inhibit HBV replication, primarily by restricting the transcription of cccDNA (Zhao et al., 2022; Wei and Meng, 2023). Strikingly, IFN-α2b can inhibit this downregulation of HDAC3, controlling the transcription and replication of HBV through facilitating the histone H4K8 de-2-hydroxyisobutyrylation caused by HDAC3 (Zhao et al., 2022). Contrary to HBV, HCV activates HDACs with subsequent acetylation of histones via inducing ROS (Zhou et al., 2018), which means HCV increases the expression of HDACs. The proviral role of HDACs in HCV replication has been experimentally demonstrated. Specifically, treatment with an HDAC3 inhibitor significantly suppressed HCV replication in a murine infection model, providing direct evidence for HDAC3’s involvement in facilitating viral propagation (Zhou et al., 2018). Similarly, the HDAC3 inhibitor RGFP966 has been shown to decrease viral replication in Huh7 cells and in an *in vivo* model of humanized transgenic mice (Zhou et al., 2018). This is accomplished through the downregulation of Apo-A1 expression, a protein that is essential for maintaining HCV infectivity (Mancone et al., 2011). This process may potentially lead to the suppression of HCV secretion.

Deacetylation exerts dual regulatory effects on glucose homeostasis. First, HDAC-mediated deacetylation can preserve gluconeogenic pathways, as demonstrated by hepatic FOXO1 and FOXO3 serving as specific substrates for HDAC3. Conversely, deacetylation may also suppress glycolytic activity through distinct mechanisms. FOXO1 deacetylation confers protection against oxidative stress-mediated acute β-cell dysfunction, maintaining normal insulin biosynthesis and secretory capacity (Takigawa-Imamura et al., 2003). Beyond regulating FOXO1/3 acetylation status, HDACs additionally suppress TNF-α-driven inflammatory responses. Comparative studies reveal elevated histone H3 acetylation at TNF-α promoter regions in circulating monocytes from diabetic patients versus non-diabetic controls, a modification that enhances inflammatory gene transcription during hyperglycemic episodes (Lu et al., 2021). Conversely, HDAC inhibition demonstrates therapeutic potential for ameliorating insulin resistance. Pharmacological HDAC inhibitors promote glucose transporter type 4 (GLUT4) translocation to the plasma membrane, consequently augmenting both basal and insulin-stimulated glucose uptake in skeletal muscle. These findings underscore the critical regulatory function of HDACs in modulating glucose metabolism, where their activity normally suppresses glucose uptake and contributes to insulin resistance pathogenesis (Takigawa-Imamura et al., 2003). Moreover, previous studies have demonstrated that glucose-stimulated insulin gene expression involves the molecular interplay between pancreatic and duodenal homeobox 1 (PDX-1) and p300 histone acetyltransferase (Mosley and Ozcan, 2003). The p300 protein, possessing intrinsic histone acetyltransferase activity, translocates to the insulin gene promoter through PDX-1 interaction. This recruitment enables local histone acetylation that potently enhances insulin gene transcription and subsequent protein synthesis. Reduced HDACs activity consequently diminishes insulin expression due to impaired deacetylation mechanisms. HDAC3 inhibition exhibits dual protective effects by alleviating T2DM-induced endothelial damage and suppressing β-cell apoptosis. Importantly, HDAC3 blockade significantly attenuates ER stress associated with β-cell dysfunction (He et al., 2023). Additionally, cytokine-mediated pathways combined with impaired NF-κB transactivation collectively exacerbate β-cell apoptosis and functional impairment (Weidemann et al., 2024).

Histone acetyltransferases (HATs) represent the enzymatic counterpart to HDACs in maintaining histone acetylation homeostasis. HAT1-mediated acetylation of nascent histones cooperates with chromatin assembly factors such as chromatin assembly factor-1 (CAF-1) and anti-silencing factor 1 to orchestrate nucleosome deposition. This acetylation-dependent mechanism additionally participates in fundamental nuclear processes spanning DNA replication, transcriptional regulation, damage repair, and chromatin silencing/activation pathways (Hu et al., 2023; Ortega et al., 2024). HATs catalyze lysine residue acetylation on histones, increasing chromatin accessibility for transcription factors, transcriptional machinery, and RNA polymerase II to facilitate gene activation. Emerging evidence indicates that HATs, along with their counterpart HDACs, critically regulate HBV infection. Specifically, the acetylation status of H3/H4 histones bound to cccDNA directly modulates viral replication efficiency (Pollicino et al., 2006). This mechanistic model is supported by elevated expression of HAT1, CAF-1, and hepatocellular carcinoma-associated long non-coding RNA in HBV-infected humanized liver mouse models. Notably, CAF-1 deficiency, specifically its p150 subunit, impairs histone H3/H4 deposition onto HBV cccDNA (Yang et al., 2019). These findings establish that the HAT1/CAF-1 axis mediates HBV cccDNA minichromosome formation through histone H4 lysine 5 and 12 acetylation, while maintaining viral chromatin transcriptional activity (Yang et al., 2019).

Although direct evidence linking HATs to glycemic control remains limited, current studies confirm acetylation’s critical involvement in insulin secretion pathways (Zhang et al., 2019) and gluconeogenic regulation (Mutlu et al., 2024), including insulin signal transduction modulation. Future investigations should prioritize the intersection between viral hepatitis and diabetic acetylation networks, particularly the dual regulatory functions of HDACs and HATs in viral persistence and glucose homeostasis. Therapeutic exploration of HDAC inhibitors for concurrent antiviral and insulin-sensitizing effects may yield novel combination therapies. Given HAT1/CAF-1’s established roles in chromatin biology, further mechanistic studies should evaluate their potential impact on diabetic progression and assess their viability as therapeutic targets.

## 5 Conclusions

**Figure 3:**
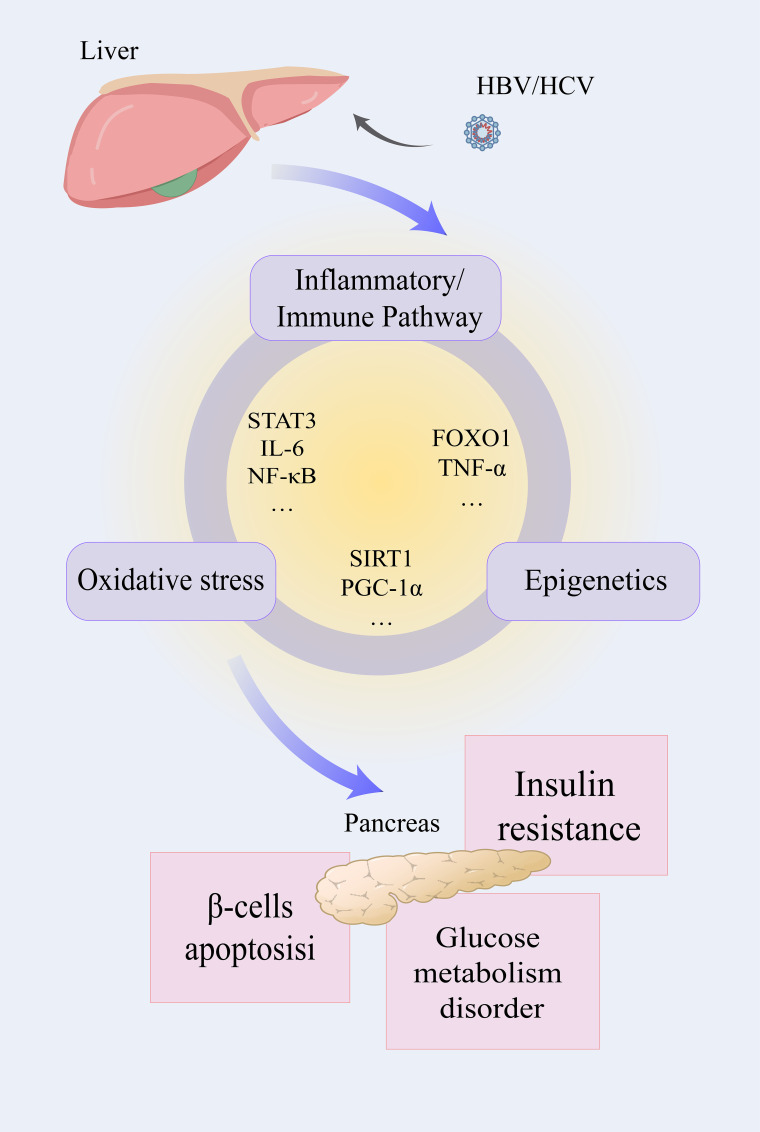
The liver infected with HBV/HCV influences the normal function of the pancreas through three interrelated pathways. After infection with the hepatitis virus, the inflammatory pathway, oxidative stress, and epigenetic modification will be activated. Inflammatory factors such as STAT3, IL-6, NF-κB, and others play a role in both the development of inflammation and oxidative stress. Oxidative stress and epigenetic modification are connected by SIRT1 and PGC-1α, and so on. FOXO1 and TNF-α affect the inflammatory pathway and epigenetic modification at the same time. Affected by the above factors, the related functions mediated by the pancreas may be influenced, and insulin resistance, glucose metabolic dysfunction, and β-cell apoptosis may occur.

Of course, these important pathways involved in the occurrence and development of diseases do not exist independently, and they are inextricably linked (**Figure 3**). STAT3, which plays a significant role in the inflammatory pathway, can also exacerbate the production of ROS. Pro-inflammatory factors are not only important participants in the development of inflammation but also serve as a crucial bridge between inflammation and oxidative stress processes. For instance, TNF-α can regulate epigenetic modifications to affect the transcriptional activity of pro-inflammatory genes. PGC-1α, regulated by epigenetic modifications, plays a central regulatory role in the oxidative stress process. These interconnected pathways form an elaborate regulatory network, where modulation of any single component induces systemic perturbations. Such complexity demands that researchers adopt both holistic and mechanistic approaches to fully elucidate the pathophysiological interactions.

HBV hepatitis B virus, HCV hepatitis C virus, IL-1β/6 interleukin-1β/6, STATD3 signal transducer and activator of transcription 3, NF-κB nuclear factor kappa-B, FOXO1 forkhead box O1, PGC-1α peroxisome proliferator-activated receptor-γ coactivator-1α, SIRT1 silent information regulator 1.

Viral hepatitis exerts systemic impacts beyond hepatic pathology, disrupting multiple organ functions and metabolic networks. In HBV-related cirrhosis patients, gut microbiota dysbiosis amplifies systemic inflammation and lipid metabolic disturbances through gut-liver axis interactions, potentially impairing insulin-mediated glucose regulation (Shi et al., 2025). Similarly, HCV infection alters intestinal microbiota composition, though direct-acting antiviral (DAAs) therapy demonstrates the capacity to restore microbial balance and reduce circulating pro-inflammatory mediators in cirrhotic patients (Laivacuma et al., 2025). While these extrahepatic manifestations warrant investigation, the present review focuses specifically on hepatitis virus-induced perturbations of hepatic-pancreatic glucose regulatory pathways.

This synthesis delineates both established and emerging mechanistic links between viral hepatitis and diabetes mellitus, providing a framework for developing co-management strategies. The classical STAT3 inflammatory pathway merits deeper exploration, particularly its intersection with insulin signaling through IRS protein stabilization and β-cell preservation. The dual role of TRAIL in viral pathogenesis and glucose metabolism presents another promising research axis. Oxidative stress, primarily mediated through ER stress and NOX activation, emerges as a pivotal pathological nexus. Although research has advanced this field, important mechanisms remain unclear, particularly epigenetic changes that influence diabetes development in hepatitis patients.

Clinically, these findings underscore the necessity for vigilant glycemic monitoring in viral hepatitis patients, given their elevated risks for both metabolic complications and hepatocarcinogenesis. Therapeutic interventions should address concurrent inflammatory control and tumor surveillance. Pharmacologically, targeting ROS generation and epigenetic regulators (e.g., small-molecule modulators of histone modifications) offers therapeutic potential. However, it is crucial to acknowledge the dual nature (or: bidirectional effects) of epigenetic modifications. Consequently, when screening for therapeutic targets, the potential beneficial and adverse effects must be carefully weighed. Optimal drug development requires integrated consideration of multi-pathway effects, aiming to simultaneously ameliorate viral persistence and metabolic dysfunction while minimizing off-target consequences. Such approaches will advance precision medicine strategies for this high-risk patient population.

## Glossary

Akt/PKB: Protein kinase B
Bcl2: B-cell lymphoma/leukemia 2
CAF-1: Chromatin assembly factor-1
cccDNA: covalently closed circular DNA
C/EBPα: CCAAT enhancer binding protein α
CREB: cAMP-response element binding protein
CXCL1/14: C-X-C motif ligand 1/14
DAAs: direct-acting antivirals
DNMTs: DNA methyltransferases
ER: Endoplasmic reticulum
ERK: Extracellular regulated protein kinases
Fbw8: F-Box and wd repeat domain containing 8
Fgf21: Fibroblast growth factor 21
FoxO1: Forkhead box O1
G6Pase: Glucose-6-phosphatase
GLUT4: Glucose transporter type 4
HATs: Histone acetyltransferases
HBcAb: Hepatitis B core antibody
HBeAg: Hepatitis B virus e antigen
HBsAg(HBs): Hepatitis B surface antigen
HBV: Hepatitis B virus
HBx: Hepatitis B virus X protein
HCC: Hepatocellular Carcinoma
HCV: Hepatitis C virus
HDACs: Histone deacetylases
IFN-γ: Interferon gamma
IL-1β/2/4/6: Interleukin-1β/2/4/6
IR: Insulin resistance
IRS: Insulin receptor substrate
JAK: Janus kinase
JNK: C-Jun N-terminal kinase
MAMs: Mitochondria-associated ER membranes
mTORC1/2: Mechanistic target of rapamycin complex 1/2
NAFLD: Non-alcoholic fatty liver disease
NF-κB: Nuclear factor kappa-B
NK cell: natural killer cell
Nox: NADPH oxidases
Nrf2: Nuclear factor erythroid 2 (NF-E2)-related factor 2
NS3/5A/5B: Non-structural Protein 3/5A/5B
p38 MAPK: P38 Mitogen-Activated Protein Kinases
Pdx-1: Pancreatic and duodenal homeobox 1
PEPCK: Phosphoenolpyruvate carboxykinase
PGC-1α: Peroxisome proliferator-activated receptor-γ
PI3K: Phosphatidylinositol 3-kinase
PPAR-*γ*: Peroxisome proliferator-activated receptor gamma
PTPN13: Protein tyrosine phosphatase non-receptor 13
RAGE: Receptor of advanced glycation endproducts
RANKL: Nuclear factor kappa-B ligand
ROS: Reactive oxygen species
SIRT1: silent information regulator 1
SOCS: Suppressors of Cytokine Signaling
STAT: Signal Transducer and Activator of Transcription
T2DM: Type 2 diabetes mellitus
TLR4: Toll-like receptor 4
TNF-α: Tumor necrosis factor Alpha
TRAIL: TNF-related Apoptosis-Inducing Ligand
TRAILR1: TRAIL Receptor 1
UBL: Ubiquitin-like protein.
